# Relationship between the main components of the crystalline lens and the anterior chamber depth after cataract formation

**DOI:** 10.1007/s00417-023-06080-7

**Published:** 2023-04-28

**Authors:** Cecilia Díez-Montero, Alberto López-de la Rosa, Alberto López-Miguel, Miguel J. Maldonado

**Affiliations:** 1 Departamento de Oftalmología, Complejo Asistencial de Ávila (Ávila) y Hospital del Río Hortega (Valladolid), Ávila, Spain; 2 Instituto de Oftalmobiología Aplicada (IOBA), Universidad de Valladolid, Paseo de Belén 17, 47011 Valladolid, Spain; 3 Red Temática de Investigación Colaborativa en Oftalmología (OftaRed), Instituto de Salud Carlos III, Madrid, Spain

**Keywords:** Lens thickness, Anterior cortex, Posterior cortex, Nucleus thickness, Anterior chamber depth, Axial length, Cataract

## Abstract

**Purpose:**

To assess the relationship between anterior chamber depth (ACD) and lens thickness (LT), as well as its three main components (anterior and posterior cortex and nucleus thickness), in cataractous and non-cataractous eyes, depending on the axial length (AxL).

**Methods:**

Anterior and posterior cortex and nucleus thickness of the crystalline lens, ACD, and AxL were measured using optical low-coherence reflectometry in cataractous and non-cataractous eyes. They were also classified into hyperopia, emmetropia, myopia, and high myopia, depending on AxL; thus, eight subgroups were created. A minimum sample size of 44 eyes (of 44 patients) for each group was recruited. Linear models were fitted for the whole sample and each AxL subgroup to assess if there were differences in the relationships between the crystalline lens variables and ACD, including age as a covariate.

**Results:**

Three hundred seventy cataract patients (237 females, 133 males) and 250 non-cataract controls (180 females, 70 males), aged 70.5 ± 9.4 and 41.9 ± 15.5 years, respectively, were recruited. The mean AxL, ACD, and LT for the cataractous and non-cataractous eyes were 23.90 ± 2.05, 24.11 ± 2.11, 2.64 ± 0.45, and 2.91 ± 0.49, 4.51 ± 0.38, 3.93 ± 0.44 mm, respectively. The inverse relationship of LT, anterior and posterior cortex, and nucleus thickness with ACD was not significantly (*p* ≥ 0.26) different between cataractous and non-cataractous eyes. Further subclassification of the sample depending on AxL showed that the inverse relationship between the posterior cortex and ACD was no longer significant (*p* > 0.05) for any non-cataractous AxL group. LT, anterior and posterior cortex, and nucleus thickness was not significantly (*p* ≥ 0.43) different between cataractous and non-cataractous eyes for the whole sample, and all AxL groups after adjusting for age.

**Conclusions:**

The presence of cataracts does not modify the inverse relationship of the LT, anterior and posterior cortex, and nucleus with ACD. And this relationship does not seem to depend importantly on AxL. Besides, the possible differences in LT, anterior and posterior cortex, and nucleus between cataractous and non-cataractous eyes may not be caused by lens opacification, but possibly by the progressive lens growth due to aging.

**Supplementary Information:**

The online version contains supplementary material available at 10.1007/s00417-023-06080-7.

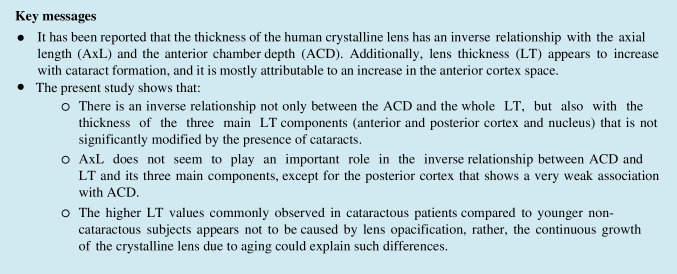



## Introduction

Precise calculations of pseudophakic intraocular lens (IOL) power in cataractous eyes was better achieved once optical biometers and new generation IOL power formulas became commercially available [1–4]. Latest generation formulas have incorporated further anterior segment parameters, including lens thickness (LT) [5], which allowed for increasing the accuracy of the IOL power calculation.

Previous authors have reported normative data for lens thickness (LT), especially in cataract surgery candidates [6–10]. It has been observed that LT increases with age in the adult population, thus a decrease in anterior chamber depth (ACD) should be also expected [7, 11]. Actually, LT is the main factor that affects ACD, followed by AxL [1]. In fact, it has been widely reported in the cataractous lens that the increase in LT results in a narrower ACD [9, 10, 12].

Optical low-coherence reflectometry (OLCR) allows precise measurement of the three main components of the crystalline lens: the anterior and posterior cortical distances and the nucleus thickness [13]. Analyzing the location and thickness of these three components can be clinically useful because it has been reported that the intracrystalline interphase point (the interphase between the anterior cortex distance and the nucleus of the lens measured with OLCR technology) can successfully increase the prediction of the final IOL position after cataract surgery [14].

Shammas et al. [13] observed that the increase in LT due to cataracts can be mostly attributable to a change in the anterior cortex distance (as measured with OLCR technology). Moreover, these authors found an inverse relationship between the three main lens components and ACD. However, LT decreases with longer axial length (AxL), regardless of the ethnicity [7, 15, 16]. Unfortunately, this indirect correlation between LT and AxL seems not to be linear for all AxL magnitudes because this relationship might change, becoming even the opposite (direct relationship) for very short and long eyes with cataract [12]. These findings show that the magnitude of AxL must be always considered when assessing the relationship among eyeball parameters to better characterize ocular anatomy [17]. In addition, Shammas et al. [13] did not consider the relationship between age and LT in their study. Thus, their outcomes could be biased because age could have acted as a confounding factor.

Consequently, the aim of the present study was to assess the relationship between the three main components of LT and ACD in cataractous and non-cataractous eyes in different AxL groups.

## Methods

This prospective cross-sectional study was approved by the local Ethics Committee and was conducted in accordance with the Declaration of Helsinki guidelines. Written consent was obtained from all subjects after explanation of the study protocol.

### Participants

Volunteers were invited to participate if they were over 18 years of age. The study population was initially classified into two main groups, cataractous and non-cataractous eyes. The criteria for being included in the cataract group was patients seeking eye care due to a visually significant cataract in both eyes attending the outpatient consultation, and grade ≥ 1 for cortical or posterior subcapsular cataract and/or grade ≥ 2 for nuclear opacities [18], according to Lens Opacification Classification System (LOCS)-III system [19]. The non-cataractous group was mainly recruited from the same ophthalmology outpatient clinic among patients who were seeking eye care for regular examination, and also volunteers from the hospital and local university staff. These subjects were not detected to have any anomaly during the outpatient examination and were invited to participate in the study. If they complied with the inclusion and exclusion criteria, they were finally included in the non-cataract group. In addition to grouping the participants depending on the presence or absence of cataract, they were also classified into four subgroups depending on the AxL as measured with OLCR technology [20, 21]. High myopia was defined as AxL ≥ 26.00 mm, myopia as AxL 24.50–25.99 mm, emmetropia as AxL 22.00–24.49 mm, and hyperopia as AxL < 22.00 mm. Exclusion criteria were history of previous eye surgery (including refractive surgery) or ocular trauma, or an active anterior and/or posterior segment anomaly. Cataract patients were also excluded if they had a congenital or traumatic origin or had a cataract associated with exposure to radiation or toxic agents. Only one eye per patient was included in the study, following a randomization table.

### Optical biometry

**Fig. 1:**
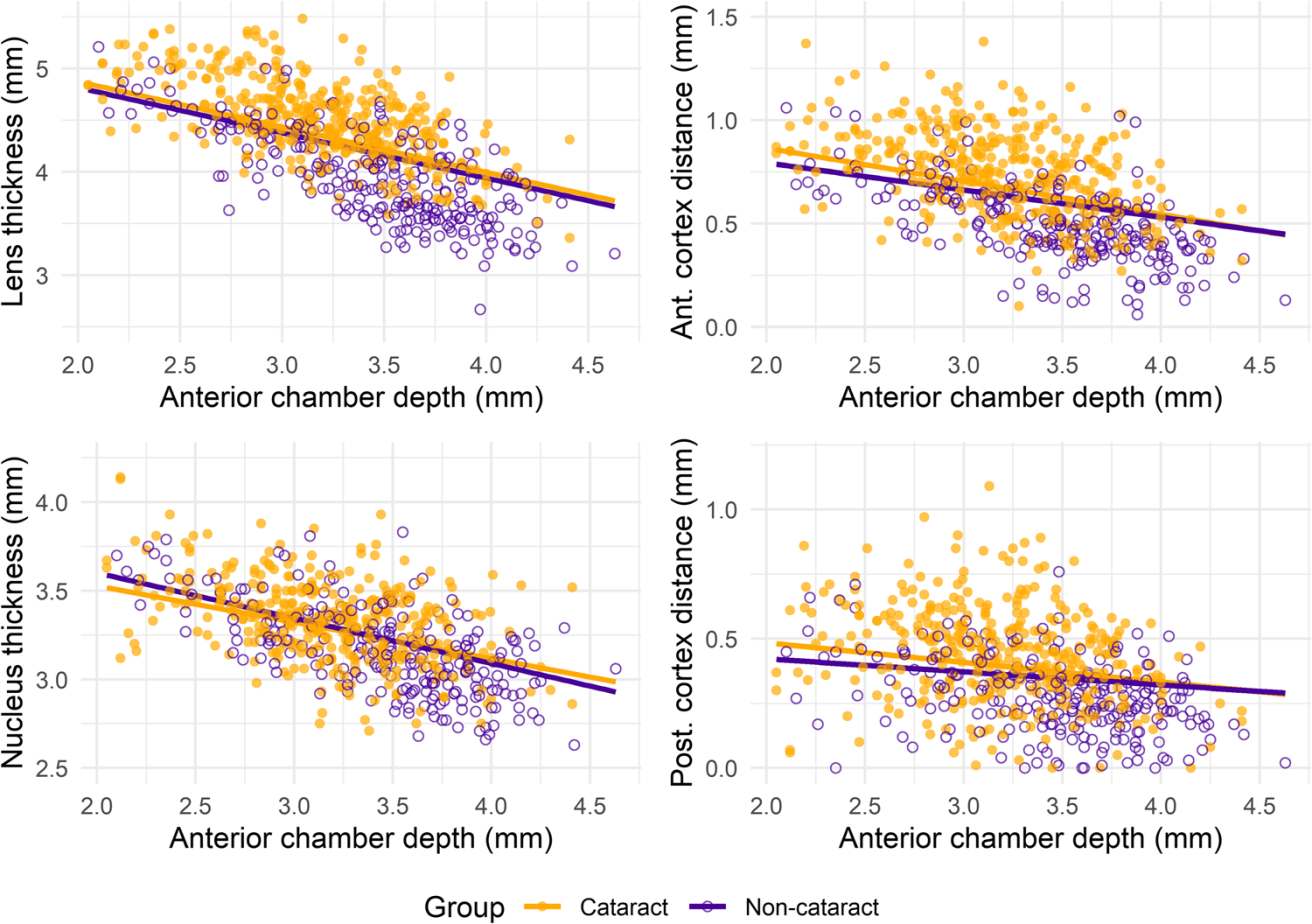
Relationship of the anterior chamber depth with the crystalline lens thickness, the anterior and posterior cortex distance and the nucleus thickness for all cataract and non-cataract eyes included. The lines represent the best-fit line for each group, considering age as a covariate

Optical biometry was performed with the Lenstar LS900 biometer (Haag-Streit AG, Köeniz, Switzerland) by a single examiner (CDM). Each participant underwent five consecutive high-quality biometry measurements. This biometry device automatically provides ACD, LT, and AxL values, among other parameters [13]. Once the measurement is performed, the device software creates a graph with cursors able to automatically identify two spikes corresponding to the anterior and posterior lens surfaces [Online Resource. Figure 1]. In addition, within the two lens spikes, two additional spikes can be identified that correspond to the anterior and posterior surfaces of the nucleus. The software also allows to move the cursors on the graph. Thus, considering that four spikes are visible for the lens, the anterior and posterior cortical distances, and the nucleus thickness, can be manually measured. Five measures were obtained for each distance and the mean was computed for analysis. The same examiner (CDM) performed the manual measurements in all eyes to avoid interobserver variability.

### Statistical analysis

Statistical analysis was performed using R statistical package version 4.0.0. Sample size was estimated using the “pwr” R package for the general linear model, considering that models would include three regression coefficients (one per independent variable), establishing a medium effect size (f^2^ = 0.15) [22], and assuming a statistical power of 80% with a level of significance of 0.05. The minimum sample size calculated for each model was 77 eyes. This calculation was for the whole sample, regardless of cataract grouping, because one of the regression coefficients included in the calculation already considered the effect of cataract grouping. Aiming both cataract groups (cataract and non-cataract) to be equilibrated, at least 39 eyes per group were considered. Axial length grouping was not considered in the sample size calculation, because different models should be adjusted. Thus, to avoid underpowered analyses, the sample size calculated (*n* = 39 eyes) was mandatory for each axial length subgroup (hyperopia, emmetropia, myopia and high myopia). Finally, adjusting for a 10% dropout rate, at least 44 eyes per each cataract and non-cataract group, considering each AxL subgroup, were included.

The participants were recruited consecutively for all groups, and the study was completed only when a sample size of at least 44 subjects was achieved in the eight groups.

The relationship between the ACD and the different lens components (LT and its three main components) was analyzed by fitting linear models using the “stats” R package. Models included the ACD as dependent variable, and the independent variables were group classification (cataract vs non-cataract), LT and its three main components (anterior and posterior cortex distances, and nucleus thickness), and the interaction among them. Age was additionally included as a covariate. These models were fitted considering the whole sample and the four individual AxL groups. The assumptions of normality, heterocedasticity, linearity, and lack of outliers were checked plotting the residuals of each fitted model. In addition, the marginal mean and slope (with 95% confidence interval) of each group were estimated and compared between groups using the “emmeans” R package. The confidence interval of the slope was also used to reveal which independent groups had a significant (confidence intervals not including the value 0) relationship. The false discovery rate was applied to control for type I errors due to multiple testing.

## Results

**Fig. 2:**
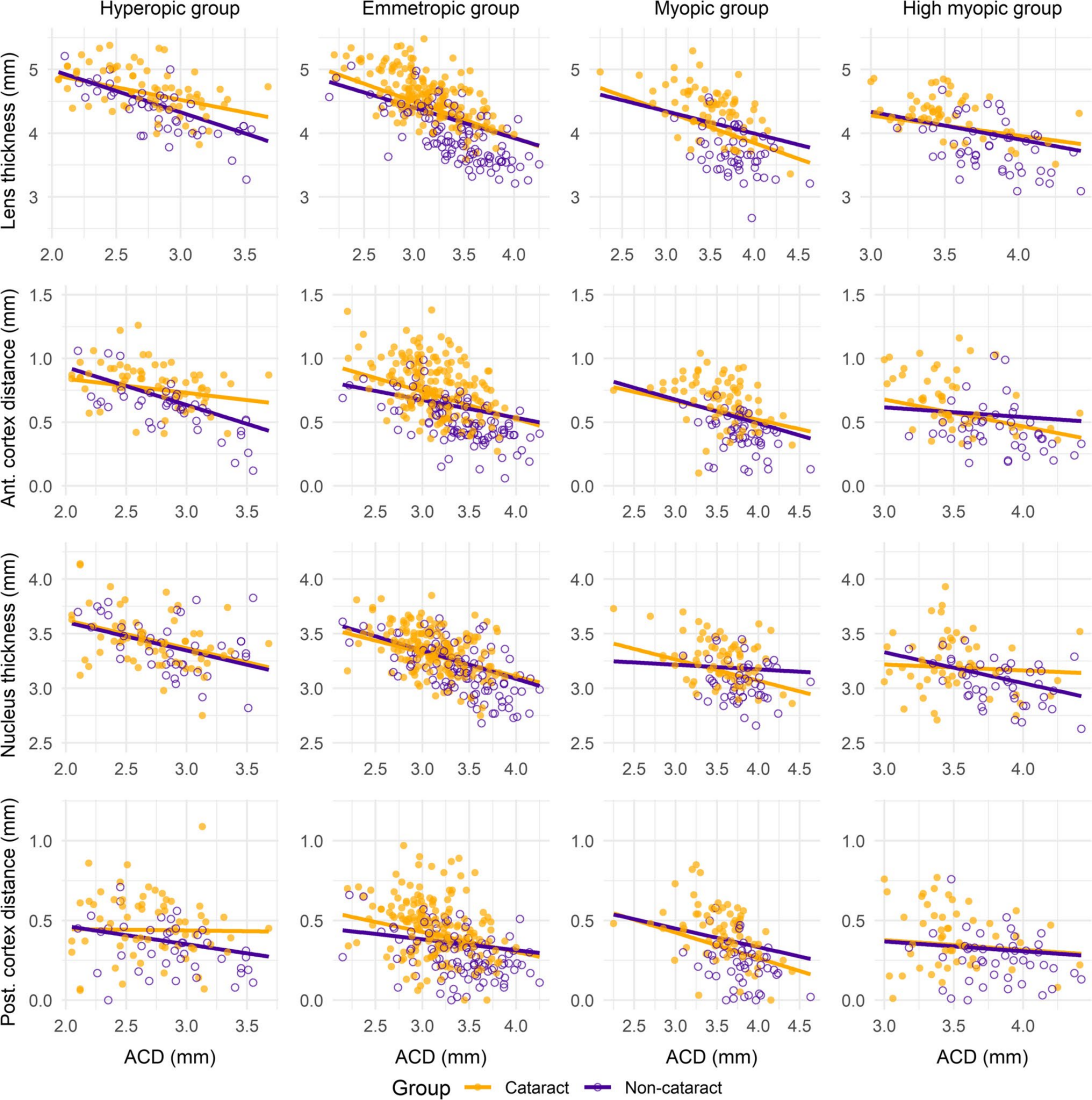
Relationship of the anterior chamber depth with the crystalline lens thickness, the anterior and posterior cortex distance and the nucleus thickness in cataract and non-cataract eyes for each axial length group. The lines represent the best-fit line for each group, considering age as a covariate

This study included 620 eyes of 620 Caucasian participants (203 men, 417 women) who were consecutively recruited. The sample was composed of 113 hyperopic (68 cataract, 45 non-cataract), 285 emmetropic (178 cataract, 107 non-cataract), 122 myopic (71 cataract, 51 non-cataract) and 100 high myopic (53 cataract, 47 non-cataract) volunteers. The mean ages of the hyperopic, emmetropic, myopic, and high myopic cataract patients recruited were 72.9 ± 8.6, 72.1 ± 8.7, 68.9 ± 9.0, and 64.5 ± 10.6 years, and for the non-cataract participants were 54.3 ± 12.7, 40.8 ± 15.3, 34.2 ± 12.8, and 41.1 ± 14.4 years, respectively [Online Resource. Figure 2]. The mean AxL for the hyperopic, emmetropic, myopic, and high myopic groups were 21.42 ± 0.51 mm, 23.27 ± 0.66 mm, 25.14 ± 0.40 mm, and 27.55 ± 1.37 mm, respectively. And the mean ACD were 2.22 ± 0.37 mm, 2.68 ± 0.39 mm, 3.09 ± 0.35 mm, and 3.11 ± 0.32 mm, respectively.

### Relationship between anterior chamber depth and lens thickness

A significant (*p* ≤ 0.004) effect of the covariate age was found for the five models (whole sample and each AxL group). The ACD showed a significant (*p* < 0.001) inverse relationship with LT for the whole sample (Fig. 1) and within each AxL group (Fig. 2). However, no significant differences were found in the mean LT between the cataract and non-cataract groups (*p* ≥ 0.43) or the interaction between LT and ACD (cataract vs non-cataract for the relationship between LT and ACD, *p* ≥ 0.54) in any of the five models (Table 1).

**Table 1:** Estimated marginal means of the main lens components (lens thickness, anterior cortex distance, nucleus thickness, and posterior cortex distance) and its slope of the relationship with the anterior chamber depth in the cataract and non-cataract groups

		Thickness / Distance (mm)			Relationship with the ACD		
		Cataract group *N* = 370 mean (95% CI)	Non-cataract group *N* = 250 mean (95% CI)	*P*-value (adjusted *p*-value)	Cataract group *N* = 370 slope (95% CI)	Non-cataract group *N* = 250 slope (95% CI)	*P*-value (adjusted *p*-value)
Lens thickness	**Whole sample**	4.30 (4.27/4.34)	4.25 (4.20/4.29)	0.09 (0.43)	**-0.44** **(-0.50/-0.38)**	**-0.44** **(-0.51/-0.36)**	0.98 (1.00)
	**Hyperopic group (*n* = 113)**	4.61 (4.53/4.69)	4.48 (4.37/4.58)	0.07 (0.43)	**-0.40** **(-0.59/-0.20)**	**-0.67** **(-0.90/-0.43)**	0.07 (0.54)
	**Emmetropic group (*n* = 285)**	4.37 (4.31/4.42)	4.29 (4.22/4.36)	0.17 (0.43)	**-0.56** **(-0.68/-0.45)**	**-0.48** **(-0.62/-0.34)**	0.33 (0.56)
	**Myopic group (*n* = 122)**	4.02 (3.94/4.11)	4.12 (4.01/4.23)	0.27 (0.46)	**-0.49** **(-0.65/-0.34)**	**-0.35** **(-0.60/-0.10)**	0.33 (0.56)
	**High myopic group (*n* = 100)**	4.07 (3.99/4.16)	4.06 (3.97/4.15)	0.79 (0.83)	**-0.31** **(-0.54/-0.09)**	**-0.43** **(-0.69/-0.17)**	0.49 (0.66)
Anterior cortex distance	**Whole sample**	0.66 (0.64/0.68)	0.62 (0.60/0.65)	0.09 (0.43)	**-0.16** **(-0.20/-0.12)**	**-0.13** **(-0.18/-0.09)**	0.31 (0.56)
	**Hyperopic group (*n* = 113)**	0.76 (0.71/0.80)	0.70 (0.65/0.76)	0.17 (0.46)	**-0.11** **(-0.21/-0.02)**	**-0.30** **(-0.41/-0.18)**	0.01 (0.26)
	**Emmetropic group (*n* = 285)**	0.69 (0.66/0.72)	0.64 (0.60/0.69)	0.15 (0.43)	**-0.21** **(-0.28/-0.14)**	**-0.14** **(-0.23/-0.05)**	0.17 (0.54)
	**Myopic group (*n* = 122)**	0.57 (0.52/0.62)	0.56 (0.49/0.62)	0.78 (0.83)	**-0.15** **(-0.24/-0.05)**	**-0.19** **(-0.34/-0.04)**	0.64 (0.79)
	**High myopic group (*n* = 100)**	0.54 (0.49/0.60)	0.57 (0.51/0.63)	0.57 (0.72)	**-0.21** **(-0.36/-0.06)**	**-0.08** **(-0.25/0.10)**	0.23 (0.54)
Nucleus thickness	**Whole sample**	3.26 (3.23/3.29)	3.27 (3.24/3.31)	0.66 (0.76)	**-0.21** **(-0.25/-0.16)**	**-0.25** **(-0.31/-0.20)**	0.18 (0.54)
	**Hyperopic group (*n* = 113)**	3.41 (3.34/3.49)	3.41 (3.32/3.51)	0.95 (0.95)	**-0.28** **(-0.45/-0.11)**	**-0.25** **(-0.46/-0.05)**	0.86 (0.95)
	**Emmetropic group (*n* = 285)**	3.28 (3.24/3.31)	3.29 (3.24/3.34)	0.79 (0.83)	**-0.22** **(-0.30/-0.14)**	**-0.26** **(-0.36/-0.17)**	0.49 (0.66)
	**Myopic group (*n* = 122)**	3.14 (3.07/3.21)	3.19 (3.10/3.28)	0.46 (0.64)	**-0.20** **(-0.32/-0.07)**	-0.04 (-0.24/0.15)	0.19 (0.54)
	**High myopic group (*n* = 100)**	3.18 (3.10/3.27)	3.15 (3.06/3.24)	0.59 (0.72)	-0.05 (-0.27/0.16)	**-0.28** **(-0.54/-0.03)**	0.17 (0.54)
Posterior cortex distance	**Whole sample**	0.39 (0.37/0.41)	0.36 (0.33/0.38)	0.14 (0.43)	**-0.08** **(-0.12/-0.04)**	**-0.05** **(-0.10/-0.01)**	0.39 (0.60)
	**Hyperopic group (*n* = 113)**	0.45 (0.39/0.51)	0.37 (0.29/0.45)	0.14 (0.43)	0.01 (-0.13/0.15)	-0.12 (-0.29/0.05)	0.24 (0.54)
	**Emmetropic group (*n* = 285)**	0.40 (0.37/0.43)	0.37 (0.32/0.41)	0.34 (0.48)	**-0.13** **(-0.20/-0.06)**	-0.07 (-0.15/0.02)	0.24 (0.54)
	**Myopic group (*n* = 122)**	0.32 (0.27/0.37)	0.37 (0.31/0.43)	0.24 (0.46)	**-0.16** **(-0.25/-0.08)**	-0.13 (-0.26/0.01)	0.67 (0.79)
	**High myopic group (*n* = 100)**	0.34 (0.28/0.40)	0.33 (0.26/0.39)	0.85 (0.89)	-0.06 (-0.21/0.09)	-0.06 (-0.24/0.12)	1.00 (1.00)

*CI* Confidence interval; *ACD* Anterior chamber depth

Bold data indicate statistically significant relationship between anterior chamber depth and the main components of the lens thickness

### Relationship between anterior chamber depth and anterior cortex distance

A significant (*p* < 0.001) effect of the covariate age was found for the five models (whole sample and each AxL group). The ACD showed a significant (*p* ≤ 0.01) inverse relationship with the anterior cortex distance for the whole sample (Fig. 1) and within each AxL group (Fig. 2). However, no significant differences were found in the mean anterior cortex distance between the cataract and non-cataract groups (*p* ≥ 0.43) or the interaction between anterior cortex distance and ACD (cataract vs non-cataract for the relationship between anterior cortex distance and ACD, *p* ≥ 0.26) in any of the five models (Table 1).

### Relationship between anterior chamber depth and nucleus thickness

A significant (*p* < 0.001) effect of the covariate age was found for the models including the whole sample, and the emmetropic and myopic groups. In contrast, no significant (*p* ≥ 0.07) effect was found for the hyperopic and high myopic groups. The ACD showed a significant (*p* ≤ 0.006) inverse relationship with the nucleus thickness for the whole sample (Fig. 1) and each AxL group, except for the high myopic group (*p* = 0.08) (Fig. 2). No significant differences were found in the mean nucleus thickness between the cataract and non-cataract groups (*p* ≥ 0.64) or the interaction between nucleus thickness and ACD (cataract vs non-cataract for the relationship between nucleus thickness and ACD, *p* ≥ 0.54) in any of the five models (Table 1).

### Relationship between anterior chamber depth and posterior cortex distance

A significant (*p* ≤ 0.002) effect of the covariate age was found for the five models (whole sample and each AxL group), except for the hyperopic group (*p* = 0.21). The ACD showed a significant (*p* < 0.001) inverse relationship with the posterior cortex distance for the whole sample (Fig. 1) and the emmetropic and myopic groups, while no significant (*p* ≥ 0.48) effect was found for the hyperopic and high myopic groups (Fig. 2). No significant differences were found in the mean posterior cortex distance between the cataract and non-cataract groups (*p* ≥ 0.43) or the interaction between posterior cortex distance and ACD (cataract vs non-cataract for the relationship between posterior cortex distance and ACD, *p* ≥ 0.54) in any of the five models (Table 1).

## Discussion

Cataract and non-cataract participants in this study showed an inverse relationship between LT and ACD, regardless of the AxL (Fig. 1). Moreover, the slope of the correlation between LT and ACD did not change significantly after cataract formation for any AxL group (Table 1). Previous authors have also reported the presence of an inverse relationship between LT and ACD in adults and in cataractous eyes in different ethnic groups [12, 13, 18, 23, 24]. However, most of the studies only assessed cataractous eyes, thus, it was not previously reported how this inverse relationship varies after cataract formation. Consequently, this study provides evidence showing that the inverse relationship between LT and ACD is not likely to change after cataract formation.

Besides, no significant differences were observed in the LT between the cataract and non-cataract group after eliminating the role of age as a confounding factor (Table 1). These outcomes showed that LT in cataractous eyes should not be expected to be thicker in comparison with non-cataractous eyes, regardless of the AxL. Thus, LT might not depend on the presence or absence of cataracts. In fact, previous authors have reported the absence of correlation between LT and lens density in moderate cataracts [25]. In contrast, LT is more likely to depend on age [7, 12, 26, 27] and AxL, as previously reported [7, 15, 16]. The outcomes of the present study also showed that the LT tended to decrease with increasing AxL (Table 1). And this tendency might not change much in cataractous eyes. This outcome highlights the importance of classifying subjects depending on the AxL when assessing the relationship among ocular parameters. This classification based on AxL allowed a better characterization of the crystalline lens before and after cataract formation.

The anterior and posterior cortex distance and the nucleus thickness showed an inverse relationship with ACD for both cataract and non-cataract groups (Table 1 and Figs. 1). Previous authors have already reported this inverse relationship [13], however, they pooled all participants in the same group without considering either the presence or absence of cataracts or the AxL of the participants recruited. Regarding the anterior cortex distance, all cataract and non-cataract AxL groups showed a significant inverse relationship with ACD (Table 1). Likewise, in case of nucleus thickness, all groups showed a significant inverse relationship except for the non-cataract myopic and cataract high myopic groups. In contrast, the posterior cortex distance had no significant relationship with ACD when assessing each AxL group separately, except for the cataract emmetropic and myopic groups. And the relationship for both cataract and non-cataract groups after pooling all AxL groups was low (slope: -0.08 and -0.05, respectively) despite being statistically significant. Previous authors have evaluated the changes in LT with increasing age using Scheimpflug slit-lamp photography in a small sample. These authors have observed a two-fold difference in the rate of growth between the anterior and the posterior cortex [28]. Thus, this can be the reason the present study found that the inverse relationship between the anterior cortex distance and ACD is stronger than the one between the posterior cortex and ACD for both cataract and non-cataract groups.

Shammas et al. [13] have reported that the increase in LT due to cataracts was mostly attributable to an increase in the anterior cortex distance. On the contrary, in the present study, no significant variation was found in thickness for the whole crystalline lens or its three main components (anterior and posterior cortex and nucleus) after comparing cataractous and non-cataractous eyes. The main reason for not finding differences in contrast to Shammas et al. [13] might be that age was considered a confounding factor in this study. In the present study, age was included as a covariate in the statistical analysis to reduce the possible bias arising when comparing older cataract and younger non-cataract participants.

The present study has limitations. First, it is a cross-sectional study assessing ocular parameters in cataract and clear lens volunteers, and the most adequate study design should be a longitudinal one where volunteers are frequently monitored. However, this type of longitudinal study should last many years until the onset of cataracts, and the compliance with the follow-up could be reduced with increasing time. Second, the OLCR device was used to evaluate the magnitude of on-axis ocular parameters, and cataracts do not necessarily develop similarly in the center and paracentral regions of the crystalline lens (i.e., cortical cataract). However, all study participants were recruited from outpatient consultation and they attended due to a visually significant cataract, thus, it was likely that the cataract was already affecting the central area of the crystalline lens. Finally, the OLCR device measures optical path lengths that are automatically converted into geometrical lengths, considering the ocular refractive indices. The lens equivalent refractive index could change depending on cataract density [29]. Thus, the measurements observed could have also been influenced by this factor. However, the differences in LT observed between cataract and non-cataract groups were not significant (Table 1) and were—in most of the cases—lower than the possible bias produced by an increase in the phase refractive index of the lens [30].

In conclusion, there is an inverse relationship between the ACD and LT, as well as the thickness of its three main components, in both cataractous and non-cataractous eyes. The weakest association corresponds to the posterior cortex, and it can even disappear when eyes are divided based on its AxL. Besides, the increased LT typically observed in cataractous eyes in comparison with non-cataractous eyes is not likely to be caused by the presence of lens opacities, but to the continuous growth of the crystalline lens due to aging. Finally, future longitudinal studies should address precisely how the crystalline lens changes over a life span.

### Supplementary Information

Below is the link to the electronic supplementary material.

Supplementary file1 (PDF 334 kb)

## Data Availability

The data that support the findings of this study are available from the corresponding author upon reasonable request.
