# Supplementary material for: Relationship between the main components of the crystalline lens and the anterior chamber depth after cataract formation
Source: Graefes Arch Clin Exp Ophthalmol. 2023 Apr 28;261(10):2853–61. doi: 10.1007/s00417-023-06080-7 (PMC10543629; doi:10.1007/s00417-023-06080-7)
Supplement: Supplementary file 1 — Supplementary file1 (PDF 334 kb) [file 417_2023_6080_MOESM1_ESM.pdf]

ONLINE RESOURCES

Graefe's Archive for Clinical and Experimental Ophthalmology

Relationship between the Main Components of the Crystalline Lens and the Anterior Chamber Depth after Cataract Formation

Authors: Cecilia Díez-Montero, Alberto López-de la Rosa, Alberto López-Miguel, Miguel J. Maldonado.

Corresponding author: Alberto López Miguel. IOBA, Universidad de Valladolid, Valladolid, Spain.

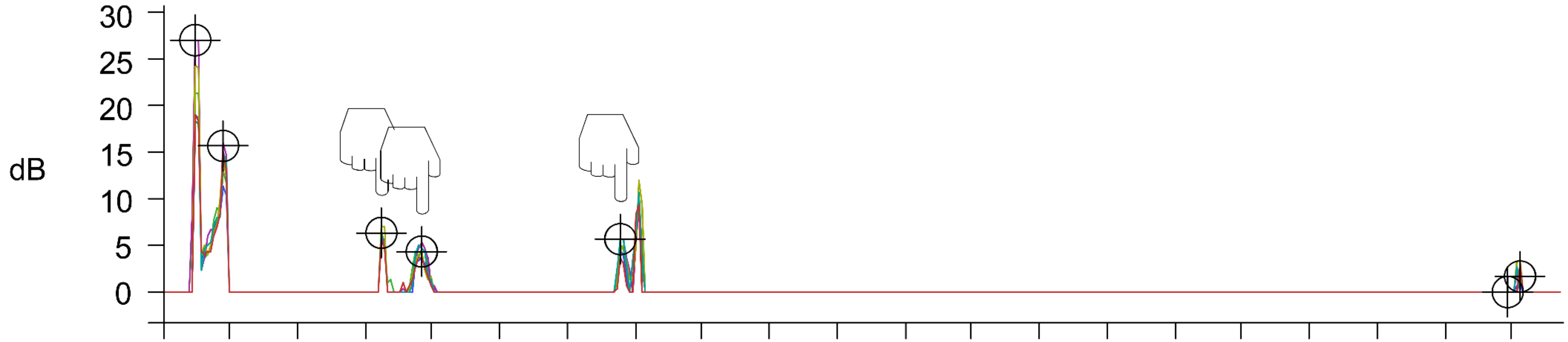

Figure 1. Graph showing the axial length measurement performed by an optical low-coherence reflectometry system (Lenstar LS900).

The anterior cortical distance and the nucleus thickness are manually measured using the cursor of the biometry system.

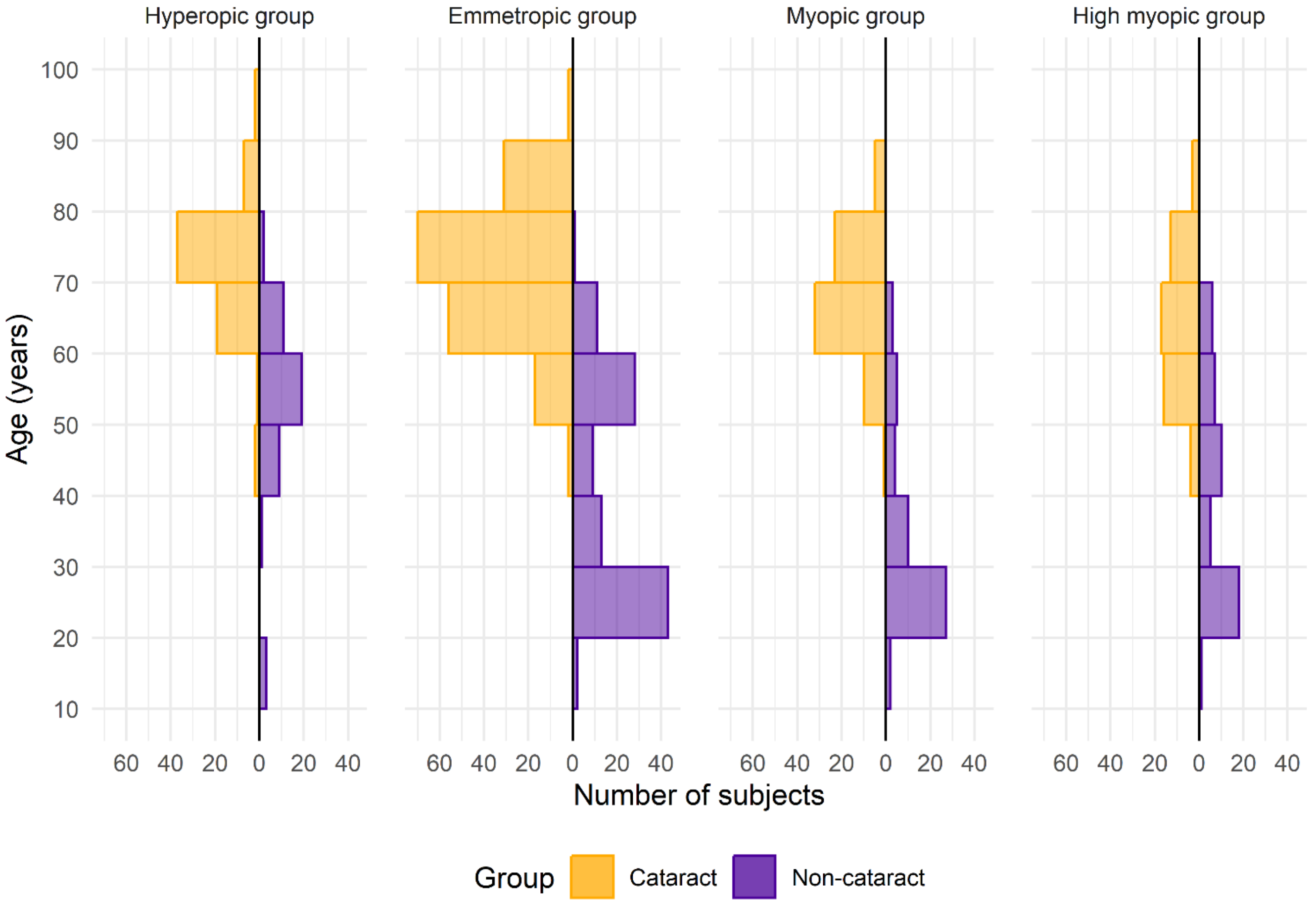

Figure 2. Distribution of the age of the participants included in the study.
